# Testosterone and Estradiol as Novel Prognostic Indicators for HBV-Related Acute-on-Chronic Liver Failure

**DOI:** 10.3389/fmed.2021.729030

**Published:** 2021-09-08

**Authors:** Shuning Sun, Baoyan Xu, Wenting Tan, Xiaomei Xiang, Yi Zhou, Yunjie Dan, Yanzhi Guo, Zhaoxia Tan, Guohong Deng

**Affiliations:** Department of Infectious Diseases, Southwest Hospital, Third Military Medical University (Amy Medical University), Chongqing, China

**Keywords:** ACLF (acute-on-chronic liver failure), hepatitis B, mortality, prognosis, testosterone (androgen), estradiol

## Abstract

**Background:** HBV-related acute-on-chronic liver failure (HBV-ACLF) has a high short-term mortality and urgently needs an early warning system with simplicity and high accuracy. Previous studies show that sex hormones play potential roles in the progression of HBV-related liver diseases.

**Aims:** To explore the effect of testosterone and estradiol on the occurrence and prognosis of HBV-ACLF.

**Methods:** A prospective cohort of 300 chronic hepatitis B (CHB) patients was enrolled among which 108 were diagnosed with HBV-ACLF at admission and 20 developed to HBV-ACLF during hospitalization. We compared the level of serum testosterone and estradiol of patients with varied ACLF background, disease severity and cirrhosis conditions and analyzed the predictive ability of short-term prognosis. A novel prognostic model involving testosterone was developed and further validated in the HBV-ACLF group.

**Results:** The baseline estradiol level of HBV-ACLF group was significantly higher while testosterone was lower than that of non-ACLF group. The estradiol level increased while the testosterone level decreased as the number of organ failures increased. Testosterone had high accuracy in predicting the short-term mortality in HBV-ACLF (AUROC = 0.726) and estradiol did better in predicting the occurrence of ACLF during hospitalization (AUROC = 0.695). The novel prognostic model involving testosterone (TATIM model) was proved to have considerable prediction efficiency in HBV-ACLF cohort with or without cirrhosis.

**Conclusion:** Testosterone could be utilized as short-term prognostic indicator for HBV-related ACLF and estradiol can help to predict its occurrence. TATIM model is a novel prognostic model for HBV-related ACLF with simplicity and good performance irrespectively of liver cirrhosis.

**Clinical Trial Registration Number:** This study was based on a sub-cohort from the prospective multicenter cohort (NCT02457637).

## Lay Summary

Sexual disparity exists in acute-on-chronic liver failure and the level of sexual hormones has significant difference between patients with and without liver failure. Testosterone and estradiol could be utilized as novel prognostic indicators irrespective of liver cirrhosis.

## Introduction

Acute-on-chronic liver failure (ACLF) is a rapid-developing clinical syndrome with a high short-term mortality (1). Hepatitis B virus ranks the first in the etiology of ACLF in East Asia and other high-HBV-prevalence regions (2, 3). HBV-related ACLF leads to approximately 120 000 deaths every year and takes up massive medical resources. Even though it has poor outcomes, timely and aggressive clinical intervention could save a considerable proportion of patients' lives (4, 5). Therefore, early warning of ACLF is necessary and urgently needed.

Previous studies have shown that males are dominated in most chronic liver diseases and liver failure (6, 7). The level of sexual hormones has significant changes in the course of end-stage liver diseases (8, 9) and is associated with prognosis of cirrhosis (10), non-alcoholic fatty liver disease (NAFLD) (11) and hepatocellular carcinoma(HCC) (12). Further studies reveal that sexual hormones affect the HBV-related liver diseases via a comprehensive way including promoting or inhibiting the HBV replication (13), affecting the expression of sex hormone receptors or related transcription factors (14) and regulating the inflammatory or protective immune response (15–17). However, most of the existing studies focus on cirrhosis, NAFLD or HCC, it remains unclear whether sexual hormones are associated with HBV-ACLF.

As for early warning of liver failure, Child-Turcotte-Pugh score, Model for End-Stage Liver Disease (MELD) (18, 19), the integrated MELD (iMELD) and Sequential Organ Failure Assessment (SOFA) are the most common prognostic models for severe liver diseases but they lack the pertinence for ACLF. The Chronic Liver Failure Consortium ACLF score (CLIF-C ACLF) proposed by European Association for the Study of the Liver(EASL) in 2013 is proved to have superior predictive performance for short-term mortality in the Consortium Acute-On-Chronic Liver Failure in Cirrhosis (CANONIC) study (20). However, the major etiology of their cohort is differing from that of ACLF population in Asia-Pacific regions. The Chinese Group on the Study of Severe Hepatitis B (COSSH) proposed the COSSH-ACLF score in 2017, which have higher accuracy for predicting HBV-ACLF outcomes (3). Nevertheless, both CLIF-C ACLF and COSSH-C ACLF are based on complicated evaluation of organ failure and a simpler and more practicable scoring model is the most needed at present.

Based on the potential roles of sex hormone in HBV-related liver diseases and the urgent need of early-warning in HBV-related ACLF, we conducted this research to investigate whether testosterone and estradiol could be novel prognostic indicators for HBV-related ACLF. Firstly, we compared the level of serum testosterone and estradiol of patients with varied ACLF background, disease severity and cirrhosis conditions and then analyzed their predictive ability respectively. Finally, we used OPLS-DA and logistic regression to screen the candidate indicators and developed a testosterone-related prognostic model-TATIM, which was further validated and proved to be efficient.

## Materials and Methods

### Definitions

In this study, Chronic liver disease (CLD) included chronic hepatitis B (CHB), chronic hepatitis C, chronic hepatitis E, alcoholic liver disease, non-alcoholic steatohepatitis, chronic drug-induced liver disease, autoimmune liver disease, schistosomiasis, metabolic liver disease and cryptogenic liver disease. Events of acute decompensation included ascite, bacterial infection, upper gastrointestinal bleeding (UGIB), hepatic encephalopathy (HE) and hepatorenal syndrome (HRS). Acute liver injury was defined as alanine aminotransferase (ALT) >3 NL, aspartate aminotransferase (AST) >3 NL or total bilirubin (TBil) ≥2 NL.

The exclusion criteria included: (1) age>80 or <15; (2) with chronic extra-hepatic dysfunction; (3) hepatocellular carcinoma (HCC) or carcinoma found in other organs; (4) applying immunosuppressive agents; (5) pregnancy.

According to the APASL 2014 consensus (4) and the updated proposals (1, 21, 22), ACLF in this study was defined as acute hepatic insult manifesting as jaundice (total bilirubin [TBil] ≥5mg/dl) and coagulopathy (international normalized ratio [INR] ≥1.5), complicated by ascites and/or HE within 4 weeks in patients with previously diagnosed or undiagnosed CLD. The cirrhosis patients with or without a history of acute decompensation who met the above criteria were diagnosed as ACLF as well.

### Patients

This study is based on a sub-cohort of prospective multicenter ACLF cohort [CATCH-LIFE: NCT02457637 (23, 24), NCT03641872]. A total of 348 patients with CLD were prospectively enrolled for acute decompensation or acute liver injury from January 1, 2015 to December 31, 2016 in Southwest Hospital (Chongqing, China). Data including demographic information, clinical procedures, laboratory tests, imaging tests, hospitalization records at admission and follow-up information was collected by electronic case report form (CRF).

According to the etiological data, 300 patients were selected as CHB cohort, among which 108 patients were diagnosed with ACLF at admission while 192 were non-ACLF. Moreover, during hospitalization 20 patients progressed into ACLF while 172 did not until discharge. The flow chart of enrollment is shown in Supplementary Figure 1.

### Sexual Hormone Testing

The baseline level of serum testosterone and estradiol were tested using Cobas Testosterone II kits and Cobas Estradiol III kits on the Roche Elecsys 2010 Electrochemical Luminescent immune Analyzer (Roche Diagnostics, Rotkreauz, Switzerland).

### Model Calculation

The Child-Turcotte-Pugh (18), MELD, iMELD (19), SOFA, CLIF-C ACLF (20) and COSSH ACLF (3) scores were calculated using the baseline data. All the prognostic models were described in the references previously.

### Statistical Analysis

In this study, statistical analysis was conducted using IBM SPSS Statistics (ver. 22.0; SPSS Inc.), MedCalc(ver.19.0; MedCal Software) and SIMCA(ver.14.1;Umetrics AB). Two-tailed *P* values < 0.05 were statistically significant.

Quantitative variables are presented as mean ± SD or median (IQR) and categorical variables are presented as percentage. Differences between groups were compared by Student's *t* test, Mann-Whitney *U* test or one-way ANOVA for continuous variables and χ 2-test for categorical variables. For candidate parameters, orthogonal partial least squares discriminant analysis (OPLS-DA) was conducted to compare their predictive ability of short-term prognosis in HBV-ACLF cohort (22, 25). Multivariate logistic regression was conducted to identify the independent risk factors of 28 day mortality using the backward LR method, with entry and removal probabilities of 0.05 and 0.10, respectively. The TATIM prognostic model was developed by multivariate logistic regression. The Kaplan-Meier cumulative survival curves were compared with log-rank test. The area under receiver operating characteristics curves (AUROCs) were used to evaluate the predictive utility for 28 day and 90 day mortality and DeLong's tests were utilized to compare the AUROCs of varied scoring systems.

### Study Approval

This study fulfilled the principles of the 1975 Declaration of Helsinki and was approved by the Ethics Committee of our institution. Written informed consent was obtained from all patients or their legal representatives.

## Results

### Baseline Characteristics of Cohort

The baseline characteristics of the CHB cohort are summarized in Table 1. Data of the HBV-ACLF cohort (*N* = 108) and non-ACLF group (*N* = 192) are shown in Table 2 and Supplementary Table 1, respectively.

**Table 1:** Baseline characteristics of CHB cohort.

**Variables**	**Total (*n* = 300)**	**ACLF (*n* = 108)**	**non-ACLF (*n* = 192)**	***p*** **value**
**Clinical characteristics**				
Age (year)	45.0 ± 12.0	46.18 ± 11.89	44.40 ± 11.99	0.217
Male, *n* (%)	255 (85.0)	94 (87.0)	161 (83.9)	0.459
HBV reactivation (%)	65 (21.7)	15 (13.9)	50 (26.0)	0.014
Alcohol intaking, *n* (%)	24 (8.0)	9 (8.3)	15 (7.8)	0.873
Heptoxic drug, *n* (%)	17(5.7)	8 (7.4)	9 (4.7)	0.328
Fatigue, *n* (%)	14 (4.7)	8 (7.4)	6 (3.1)	0.091
UGIB, *n* (%)	6(2.0)	3 (2.8)	3 (1.6)	0.470
Cirrhosis, *n* (%)	153 (51.0)	57 (52.8)	96 (50.0)	0.644
Ascite, *n* (%)	102 (34.0)	37 (34.3)	65 (33.9)	0.943
HE, *n* (%)	2(0.7)	2 (1.9)	0 (0)	0.059
HBeAg +, *n* (%)	147 (49.0)	43 (42.2)	104 (54.2)	0.455
**Treatment data**				
Average Stay time (days)	21.49 ± 14.59	25.10 ± 18.07	19.46 ± 11.78	<0.001
Telipressin therapy, *n* (%)	4 (1.3)	4 (3.7)	0 (0)	0.016
Artificial liver support, *n* (%)	31 (10.3)	23 (21.3)	8 (4.2)	<0.001
NH3 reduction therapy, *n* (%)	24 (8.0)	21 (19.4)	3 (1.6)	<0.001
Diuretics therapy, *n* (%)	85 (28.3)	32 (29.6)	53 (27.6)	0.709
**Laboratory parameters**				
WBC (10∧9/L)	5.8 ± 4.0	7.17 ± 5.19	5.08 ± 2.82	<0.001
Neutrophil (10∧9/L)	4.19 ± 4.05	5.36 ± 4.47	3.54 ± 3.65	<0.001
Leukomonocyte (10∧9/L)	0.56 ± 2.13	0.90 ± 3.48	0.38 ± 0.46	0.122
TBil (mg/dL)	10.91 ± 9.02	18.33 ± 7.94	6.72 ± 6.57	<0.001
ALT (IU/L)	558.83 ± 749.02	631.39 ± 835.16	518.02 ± 694.90	0.209
AST(IU/L)	419.34 ± 502.22	475.67 ± 584.46	387.95 ± 448.53	0.148
AKP(IU/L)	136.42 ± 52.49	141.12 ± 55.69	134.26 ± 50.97	0.322
Alb (g/L)	32.5 ± 8.9	30.38 ± 5.08	33.62 ± 10.32	0.003
INR	1.58 ± 0.58	2.18 ± 0.53	1.24 ± 0.23	<0.001
Sodium (mmol/L)	137.07 ± 8.90	135.99 ± 6.16	137.67 ± 10.08	0.119
Kalium (mmol/L)	4.02 ± 0.57	4.06 ± 0.62	4.72 ± 9.81	0.419
Creatine (mg/dL)	0.80 ± 0.44	0.84 ± 0.57	0.77 ± 0.34	0.303
Log HBV-DNA(IU/ml)	4.14 ± 2.75	3.94 ± 2.76	4.26 ± 2.75	0.348
**Sexual hormones**				
E2 (pg/ml)	98.81 ± 82.11	144.22 ± 76.82	73.15 ± 73.60	<0.001
TT (ng/ml)	3.33 ± 3.31	1.59 ± 1.69	4.32 ± 3.58	<0.001
**Scoring systems**				
Child-Pugh	8.86 ± 1.92	10.51 ± 0.98	7.93 ± 1.69	<0.001
MELD	14.33 ± 7.76	21.30 ± 5.91	10.27 ± 5.45	<0.001
iMELD	2.80 ± 0.96	3.59 ± 0.86	2.35 ± 0.69	<0.001
SOFA	4.90 ± 2.26	7.22 ± 0.98	3.58 ± 1.63	<0.001
CLIF-C ACLF	32.22 ± 8.92	38.38 ± 8.49	28.76 ± 7.12	<0.001
COSSH ACLF	5.08 ± 0.97	5.99 ± 0.88	4.55 ± 0.52	<0.001

*AKP, phosphatase alkaline; ALT, alanine aminotransferase; AST, aspartate aminotransferase; CLIF-C ACLF, European Association for the Study of Chronic Liver Failure; COSSH ACLF, ACLF model of Chinese Group on the Study of Severe Hepatitis B; E2,estradiol; HBeAg, hepatitis B e antigen; HBV, hepatitis B virus; HE, hepatic encephalopathy; INR, international normalized ratio; MELD, Model for End-stage Liver Disease; SOFA, sequential organ failure assessment; TT, testosterone; UGIB, upper gastrointestinal hemorrhage; WBC, white blood count*.

**Table 2:** Analysis of baseline data of HBV-ACLF cohort.

**Variables**	**Total (*n* = 108)**	**28day Non-survivors (*n* = 24)**	**28day Survivors (*n* = 84)**	**Univariate analysis**		**Multivariate analysis**	
				**OR (95% CI)**	***p*** **value**	**OR (95% CI)**	***p*** **value**
**Clinical characteristics**							
Age (year)	46.18 ± 11.89	52.17 ± 10.81	44.46 ± 11.68	1.060 (1.016–1.105)	0.005	12.48 (0.932–166.9)	0.068
Male, *n* (%)	94 (87.0)	23 (95.8)	71 (84.5)	4.211 (0.522–33.966)	0.146		
Bacteria infection, *n* (%)	47 (43.5)	16 (66.7)	31 (6.9)	3.419 (1.313–8.907)	0.009	2.165 (0.722–6.491)	0.130
HBV reactivation, *n* (%)	15 (13.9)	2 (8.3)	13 (15.5)	0.497 (0.104–2.371)	0.372		
Alcohol intaking, *n* (%)	9 (8.3)	1 (4.2)	8 (8.5)	0.413 (0.049–3.478)	0.680		
Heptoxic drug, *n* (%)	8 (7.4)	3 (12.5)	5 (6.0)	2.257 (0.499–10.218)	0.280		
Fatigue, *n* (%)	8 (7.4)	2 (8.3)	6 (7.1)	1.182 (0.223–6.270)	0.844		
UGIB, *n* (%)	3 (2.8)	0 (0)	3 (3.6)		1.000		
Cirrhosis, *n* (%)	57 (52.8)	13 (54.2)	44 (52.4)	1.074 (0.432–2.669)	0.877		
Ascite, *n* (%)	37 (34.3)	5 (20.8)	32 (38.1)	0.428 (0.145–1.258)	0.116		
HE, *n* (%)	2 (1.9)	2 (1.9)	0 (0)		0.048		
HBeAg +, *n* (%)	43 (42.2)	7 (29.2)	36 (46.2)	2.091 (0.244–17.886)	0.492		
**Treatment characteristics**							
Telipressin therapy, *n* (%)	4 (3.7)	3 (12.5)	1 (1.2)		0.034		
Artificial liver support, *n* (%)	23 (21.3)	6 (25.0)	17 (20.2)		0.615		
NH3 reduction therapy, *n* (%)	21 (19.4)	10 (41.7)	11 (13.1)		0.002		
Diuretics therapy, *n* (%)	32 (29.6)	5 (20.8)	27 (32.1)		0.285		
**Laboratory parameters**							
WBC (10∧9/L)	7.17 ± 5.19	8.06 ± 3.76	6.92 ± 5.52	1.037 (0.959–1.121)	0.346		
Neutrophil (10∧9/L)	5.36 ± 4.47	6.06 ± 3.44	5.16 ± 4.73	1.040 (0.950–1.139)	0.383		
Leukomonocyte (10∧9/L)	0.90 ± 3.48	0.72 ± 0.87	0.95 ± 3.93	0.975 (0.820–1.160)	0.774		
TBil (mg/dL)	18.33 ± 7.94	22.78 ± 8.47	17.06 ± 7.34	1.099 (1.033–1.170)	0.002	3.105 (0.545–17.70)	0.091
ALT (IU/L)	631.39 ± 835.16	466.16 ± 711.37	678.60 ± 865.33	1.000 (0.999–1.000)	0.274		
AST (IU/L)	475.67 ± 584.46	427.74 ± 587.11	488.79 ± 586.57	1.000 (0.999–1.001)	0.659		
AKP (IU/L)	141.12 ± 55.69	118.75 ± 46.50	146.39 ± 56.66	0.989 (0.977–1.001)	0.074		
Alb (g/L)	30.38 ± 5.08	28.73 ± 5.53	30.86 ± 4.87	0.918 (0.837–1.008)	0.070		
INR	2.18 ± 0.53	2.38 ± 0.46	2.12 ± 0.54	2.444 (1.053–5.672)	0.033	8.791 (0.706–109.4)	0.023
Sodium (mmol/L)	135.99 ± 6.16	133.61 ± 9.20	136.68 ± 4.78	0.927 (0.861–0.998)	0.127		
Kalium (mmol/L)	4.06 ± 0.62	4.15 ± 0.77	4.03 ± 0.57	0.987 (0.918–1.061)	0.160		
Creatine (mg/dL)	0.84 ± 0.57	1.12 ± 0.93	0.76 ± 0.38	2.695 (1.110–+6.543)	0.077		
Log HBV-DNA (IU/ml)	3.94 ± 2.76	3.82 ± 2.88	3.97 ± 2.75	0.981 (0.824–1.169)	0.833		
**Sexual hormones**							
E2 (pg/ml)	144.22 ± 76.82	182.08 ± 99.21	134.06 ± 66.78	1.007 (1.001–1.013)	0.041	1.188 (0.260–5.435)	0.098
TT (ng/ml)	1.59 ± 1.69	0.80 ± 0.51	1.80 ± 1.83	0.317 (0.134–0.751)	<0.001	0.492 (0.214–1.133)	0.004
**Prognostic scoring systems**							
Child-Pugh	10.51 ± 0.98	10.71 ± 0.91	10.45 ± 1.00	1.313 (0.817–2.108)	0.262		
MELD	21.30 ± 5.91	24.29 ± 7.48	20.43 ± 5.10	1.129 (1.034–1.233)	0.004		
iMELD	3.59 ± 0.86	4.24 ± 1.04	3.41 ± 0.71	3.929 (1.921–8.035)	<0.001		
SOFA	7.22 ± 0.98	7.96 ± 1.20	7.01 ± 0.80	2.933 (1.633–5.265)	<0.001		
CLIF-C ACLF	38.38 ± 8.49	44.71 ± 5.61	36.57 ± 8.32	1.193 (1.090–1.305)	<0.001		
COSSH-ACLF	5.99 ± 0.88	6.83 ± 0.82	5.77 ± 0.78	4.615 (2.326–9.158)	<0.001		

*AKP, phosphatase alkaline; ALT, alanine aminotransferase; AST, aspartate aminotransferase; CLIF-C ACLF, European Association for the Study of Chronic Liver Failure; COSSH ACLF, ACLF model of Chinese Group on the Study of Severe Hepatitis B; E2,estradiol; HBeAg, hepatitis B e antigen; HBV, hepatitis B virus; HE, hepatic encephalopathy; INR, international normalized ratio; MELD, Model for End-stage Liver Disease; SOFA, sequential organ failure assessment; TT, testosterone; UGIB, upper gastrointestinal hemorrhage; WBC, white blood count*.

Among patients with ACLF, 87.0% were males, 52.8% had previous liver cirrhosis (diagnosed by radiological evidence, CT images or clinical evidence) and 49.0% were HBeAg-positive. The top five precipitating events were bacterial infection (43.5%), HBV reactivation (13.9%), recent alcohol intake (8.3%), hepatotoxic drug (7.4%) and fatigue (7.4%). The average stay of ACLF and non-ACLF patients in hospital were 25.10 ± 18.07 days and 19.46 ± 11.78 days respectively. For these newly-developed ACLF patients, the median time from admission to ACLF is 4 days ranged from 4 days to 28 days.

### Screening for the Best Candidate Prognostic Indicators

We performed univariate analysis, multivariate logistic regression and OPLS-DA to identify the best prognostic parameters for ACLF.

As shown in Table 2, the non-survivors within 28 days were significantly older than survivors and had higher ratio of bacterial infection (66.7 vs. 6.9%, *P* = 0.009) in terms of precipitating events. In regular laboratory tests, the non-survivors had significantly higher TBil and INR than survivors. In sexual hormone tests, the serum estradiol (E2) was significant higher in the non-survivor group, whereas survivor group had higher serum testosterone (TT) level.

Before the multivariate logistic regression, all the six factors above were taken into a linear regression analysis to disregarding the effect of multi-collinearity and all their variance inflation factors (VIF) were less than 10 with tolerance greater than 0.1. The multivariate logistic regression indicated that TT and INR were independent prognostic factors (*P* < 0.05, Table 2). Age, TBil and E2 were also suggested as potential indicator with their *p* values < 0.1.

**Figure 1:**
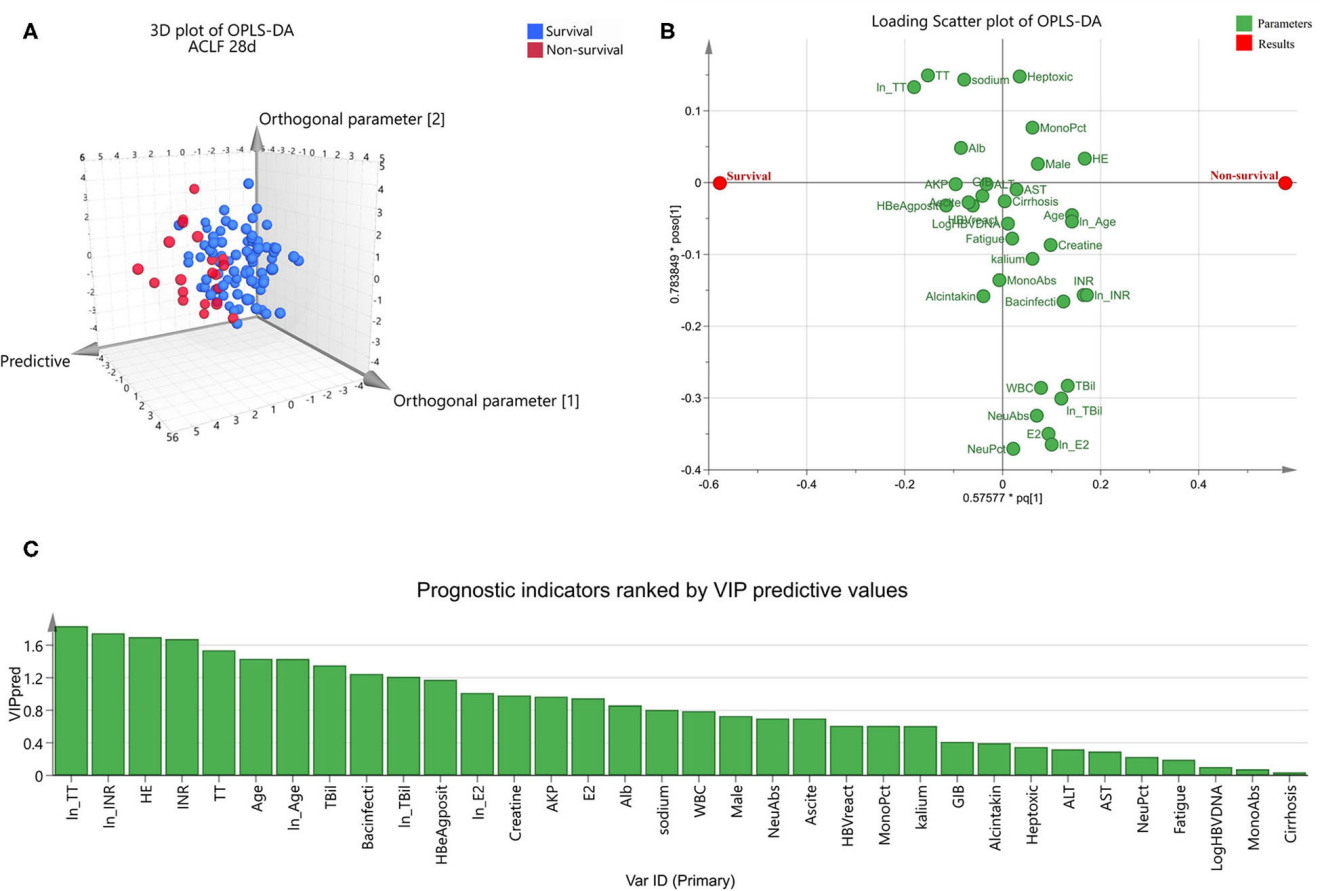
Orthogonal partial least squares discriminant analysis to identify optimal short-term prognostic parameters for hepatitis-B-related acute-on-chronic liver failure. OPLS-DA was conducted in the ACLF group (*n* = 108). Three dimensional scatter plot **(A)** distinguished non-survivors from survivors. Loading scatter plot **(B)** was used to compare the impact of each parameters on survival. Values in prediction were ranked in VIP plot **(C)**. VIPpred > 1.0 was considered to have significant contributions to the outcome.

For further exploration of candidate indicators, OPLS-DA was used to rank all their predictive ability. Three dimensional scatter plot showed that non-survivors were unambiguously distinguished from survivors (Figure 1A). Loading scatter plot (Figure 1B) evaluated the candidate variables and VIP plot (Figure 1C) ranked their importance of prediction. The top 12 parameters were ln (TT), ln (INR), HE, INR, TT, age, ln (age), TBil, Bacterial infection, ln (TBil), HBeAg positive, and ln (E2) (all VIPpred >1.0, Figure 1C).

Combing the suggestion of multivariate logistic regression and OPLS-DA, we finally selected TT, INR, age and TBil as the top four prognostic indicators.

### Impact of Testosterone and Estradiol on ACLF

Given that both univariate analysis and OPLS-DA suggested TT and E2 had remarkable predictive ability for 28 day mortality in HBV-ACLF patients, we further investigated their impact on disease severity, 28 day cumulative survival rate, 90 day cumulative survival rate and cumulative occurrence rate of ACLF during hospitalization.

**Figure 2:**
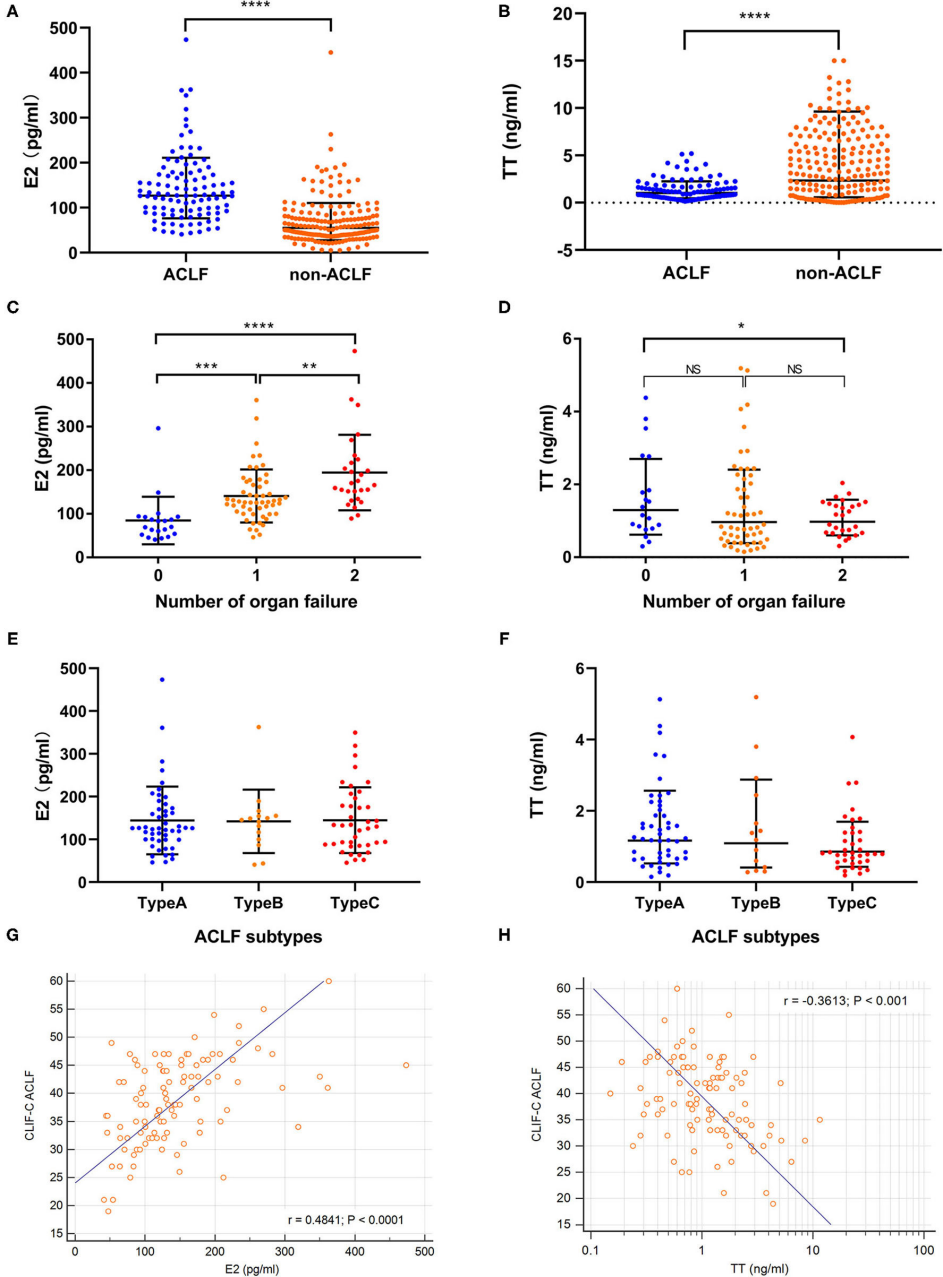
Comparison of sexual hormone levels. Comparison of sexual hormone levels were conducted in CHB cohort (*n* = 300) and HBV-ACLF group (*n* = 108). **(A,B)** Showed the baseline estradiol and testosterone levels in hepatitis B related acute-on-chronic liver failure (HBV-ACLF, *n* = 108) and non-ACLF group (*n* = 192). **(C,D)** Revealed the changes of estradiol and testosterone according to the number of organ failures. **(E,F)** Suggested that there was no significant difference of estradiol and testosterone levels among ACLF subtypes. **(G)** Showed the linear correlation between estradiol levels and traditional prognostic scores. **(H)** Showed the linear correlation between testosterone levels and traditional prognostic scores. *p < 0.05, **p < 0.01, ***p < 0.001, and ****p < 0.0001.

The baseline estradiol level of HBV-ACLF group was significantly higher while testosterone was lower than those of non-ACLF group (Figures 2A,B). As for subtypes of ACLF, there was no significant difference of sexual hormone level among type A (non-cirrhotic), type B (compensated cirrhotic) and type C (decompensated cirrhotic) (Figures 2E,F). The E2 level at admission increased while the TT level decreased as the number of organ failures increased from 0 to 2 (Figures 2C,D). The E2 level was positively correlated with MELD, SOFA and CLIF-C ACLF scores (r = 0.569, 0.593 and 0.484, all *P* < 0.001, Figure 2G) while the TT level was only negatively correlated with CLIF-C ACLF score (r = −0.3613, *P* < 0.001, Figure 2H).

**Figure 3:**
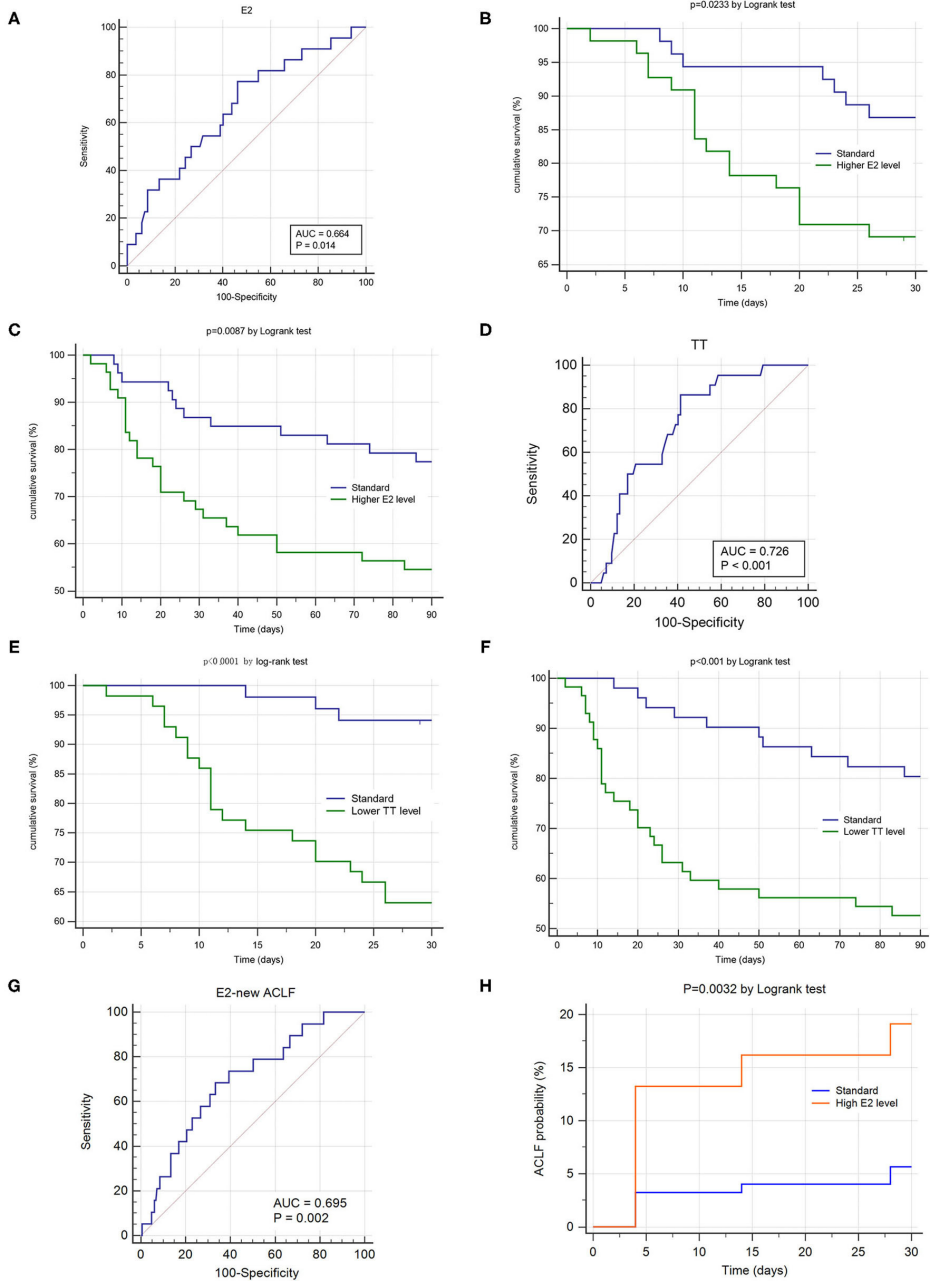
Sexual hormone's impact on prognosis and occurrence of hepatitis B related acute-on-chronic liver failure (HBV-ACLF). ROC and K-M survival analysis were conducted in the ACLF group (*n* = 108, **A–F**) and non-ACLF group (*n* = 192, **G,H**). **(A,D)** Showed the area under receiver operating characteristic cure (AUROC) of estradiol and testosterone for 28 day survival. **(B,C)** Demonstrated the differences of 28 day and 90 day cumulative survival rates according to the estradiol levels (cut-off value = 126.6 pg/ml). **(E,F)** Demonstrated the differences of 28 day and 90 day cumulative survival rates according to the testosterone levels (cut-off value = 1.16 ng/ml). **(G)** Showed the area under receiver operating characteristic cure (AUROC) of estradiol for predicting ACLF's occurrence within 28 days. **(H)** Demonstrated the differences of 28 day cumulative ACLF occurrence rate according to the estradiol levels (cut-off value = 69.79 pg/ml).

The AUROC of E2 for 28-day mortality was 0.664 (Figure 3A), with a sensitivity of 0.773 and specificity of 0.537 at a cut-off value of 126.4 pg/ml. The AUROC of TT for 28-day mortality was 0.726 (Figure 3D), with a sensitivity of 0.864 and specificity of 0.586 at cut-off value of 1.16 ng/ml.

According to their cut-off values, patients were allocated into two series: higher E2 group vs. standard E2 group and lower TT group vs. standard TT group. The 28d and 90d cumulative survival rate of the higher E2 group was significantly lower than those of standard E2 group, respectively. (*P* = 0.023, *P* = 0.009 by log-rank test, Figures 3B,C) Also, the 28d and 90d cumulative survival rate of the lower TT group was significantly lower than those of standard TT group, respectively. (*P* < 0.001, *P* < 0.001 by log-rank test, Figures 3E,F).

We conducted ROC analysis in ACLF subtype and found that the optimal TT cut-off value for Type A and Type C was both 1.16 ng/ml (Type A: *n* = 51, AUROC = 0.732, *p* = 0.009, sensitivity = 0.80, specificity = 0.667; Type C: *n* = 42, AUROC = 0.658, *p* = 0.097, sensitivity = 0.90, specificity = 0.433),while it was 0.6 ng/ml for Type B (Type B: *n* = 15, AUROC = 0.885, *p* = 0.003, sensitivity = 1.00, specificity = 0.769).

We used the prospective data of 192 non-ACLF patients at admission to evaluate the impact of E2 and TT on occurrence rate of ACLF within hospitalization. Only E2 showed significant predictive ability for occurrence of new ACLF. (*P* = 0.002, Figure 3G) The AUROC of E2 for predicting ACLF's occurrence within 28 days was 0.695, with a sensitivity of 0.684 and specificity of 0.667 at a cut-off value of 69.79 pg/ml. According to the cut-off value, the patients of non-ACLF at admission were allocated into higher E2 group and standard E2 group. The 28d cumulative ACLF occurrence rate of the higher E2 group was significantly higher than that of standard E2 group. (*P* = 0.0032 by log-rank test, Figure 3H).

Considering the definition of sub-type is derived from ACLF patients and the sample size of newly-developed ACLF is small (*n* = 20), we did not conduct E2's ROC analysis in sub-groups.

### TATIM Model's Establishment and Validations

**Figure 4:**
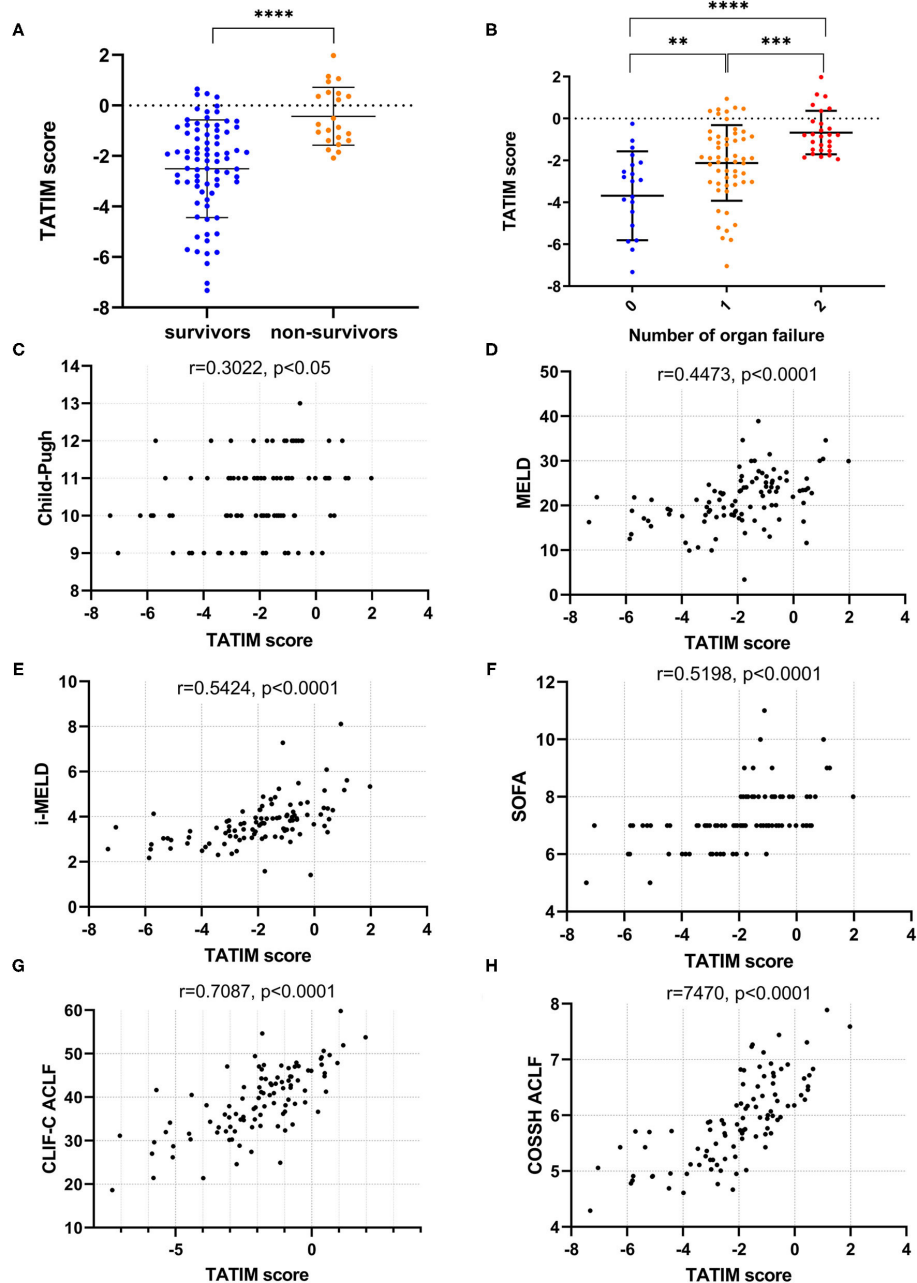
TATIM model's correlation with disease severity. Correlation analysis was conducted in the ACLF group (*n* = 108). **(A)** Showed the TATIM scores in hepatitis B related acute-on-chronic liver failure (HBV-ACLF, *n* = 108) and non-ACLF group (*n* = 192). **(B)** Revealed the changes of TATIM scores according to the number of organ failures. **(C–H)** Showed the linear correlation between TATIM scores and previous prognostic scores. **p < 0.01, ***p < 0.001, and ****p < 0.0001.

Based on the top four prognostic indicators (TT, INR, age and TBil) and Gender (male =1, female = 0), we established a novel prognostic model by multivariate logistic regression:

TATIM score = 0.056^*^TBil + 0.057^*^Age-1.148^*^TT + 0.742^*^INR + 1.963^*^Male-7.378. The TATIM score of non-survivors was significantly higher than that of survivors (−0.428 ± 1.146 vs. −2.504 ± 1.936, *P* < 0.001, Figure 4A) and the TATIM score increased as the number of organ failures increased from 0 to 2 (Figure 4B).

The TATIM score had positive correlation with the Child-Pugh, MELD, iMELD, SOFA, CLIF-C ACLF and COSSH ACLF scores (r = 0.3022, 0.4473, 0.5424, 0.5198, 7087 and 0.7470, respectively; all P <0.05; Figures 4C–H).

**Figure 5:**
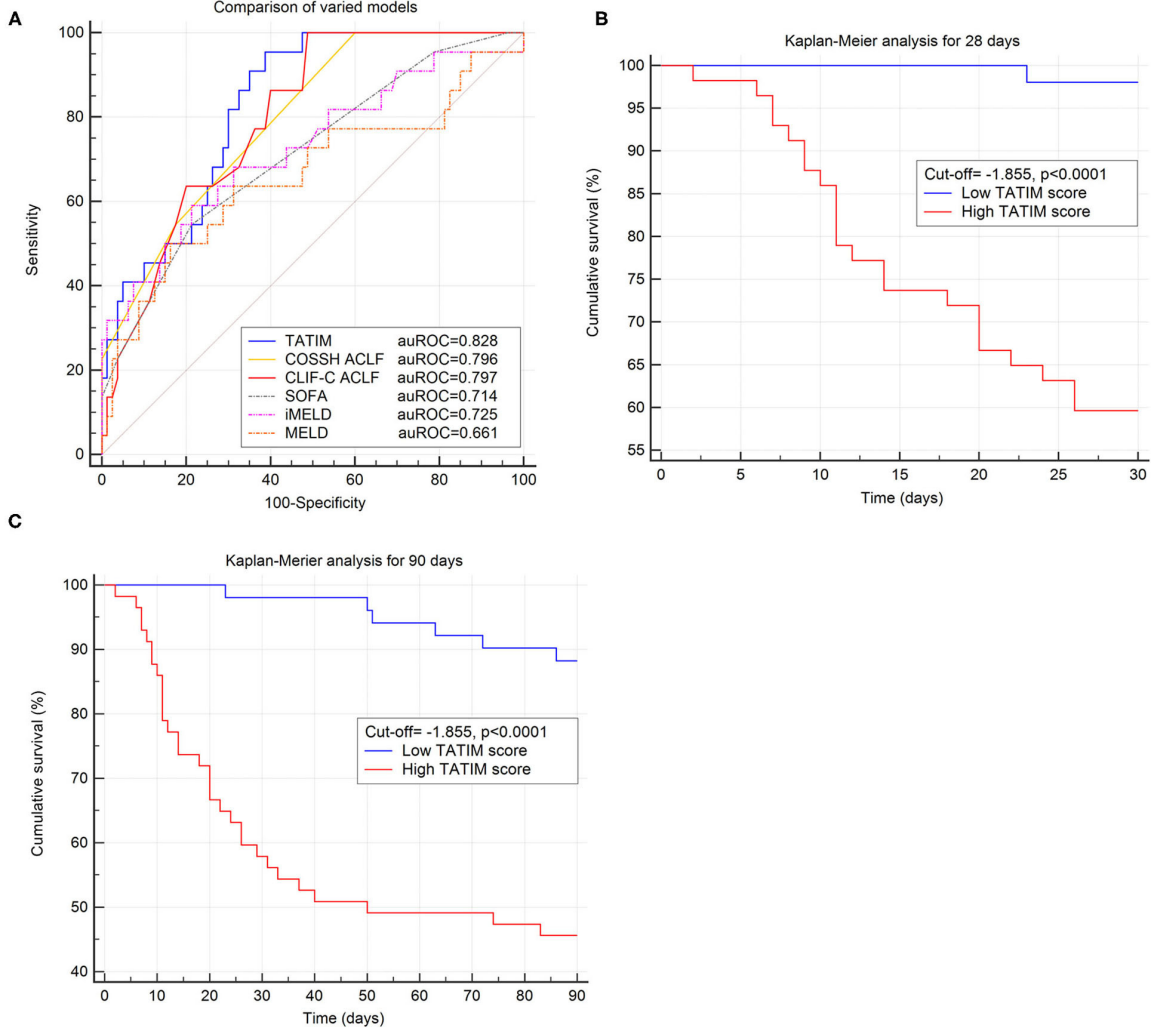
Evaluation of TATIM model in predicting the short-term prognosis of ACLF. Comparison of predictive efficiency of varied prognostic models was conducted in the ACLF group (n = 108). **(A)** Compared the area under receiver operating characteristic cure (AUROC) of TATIM model and other prognostic scores for 28 day survival. The AUROC of TATIM was 0.828 (*p* < 0.001), which was higher than MELD, iMELD, SOFA, CLIF-SOFA and comparable with CLIF-C ACLF. **(B,C)** Demonstrated the differences of 28 day and 90 day cumulative survival rates according to the TATIM score (cut-off value = −1.855).

The AUROC of TATIM score for 28-day mortality was 0.828 (*p* < 0,001), with a sensitivity of 0.955 and a specificity of 0.610 at an optimal cut-off value of −1.855. The TATIM score showed higher predictive power for 28 day mortality in HBV-ACLF patients than the MELD, iMELD and SOFA, (AUROC = 0.661, 0.725 and 0.714 respectively) and comparable predictive power with CLIF-C ACLF and COSSH ACLF score. (AUROC = 0.797 and 0.796 respectively; Figure 5A) The sensitivity and specificity of CLIF-C ACLF were 1.00 and 0.506 while those of COSSH ACLF were 1.00 and 0.405. The detailed parameters of other models were listed in Table 3.

**Table 3:** Comparison of predictive efficiency of varied prognostic models.

**Models**	**28 day prediction**						
	**AUROC**	**95%CI**	***Z*** **value**	***P*** **value**	**Sensitivity**	**Specificity**	**Cut-off**
TATIM	0.828	0.738–0.892	7.620	<0.001	95.5	60.1	−1.855
CLIF-C ACLF	0.797	0.706–0.870	6.441	<0.001	100.0	50.6	36
COSSH ACLF	0.796	0.705–0.869	7.293	<0.001	100.0	40.5	5
SOFA	0.729	0.635–0.811	4.091	<0.001	58.33	78.3	7
iMELD	0.725	0.628–0.809	4.113	<0.001	62.5	79.8	4
MELD	0.661	0.561–0.752	2.645	<0.001	54.2	84.2	25
Child-Pugh	0.547	0.446–0.646	1.280	0.201	66.7	53.6	10

*CLIF-C ACLF, ACLF model of European Association for the Study of Chronic Liver Failure; SOFA, chronic liver failure-sequential organ failure assessment; MELD, Model for End-stage Liver Disease; SOFA, sequential organ failure assessment; COSSH ACLF, ACLF model of Chinese Group on the Study of Severe Hepatitis B*.

According to the optimal TATIM cut-off value patients were divided into high-TATIM group and low-TATIM group. The 28d and 90d cumulative survival rate of high-TATIM group were significantly lower than those of low-TATIM group, respectively (*P* < 0.001 by log-rank test, Figures 5B,C).

### TATIM Score in ACLF Subtypes

**Figure 6:**
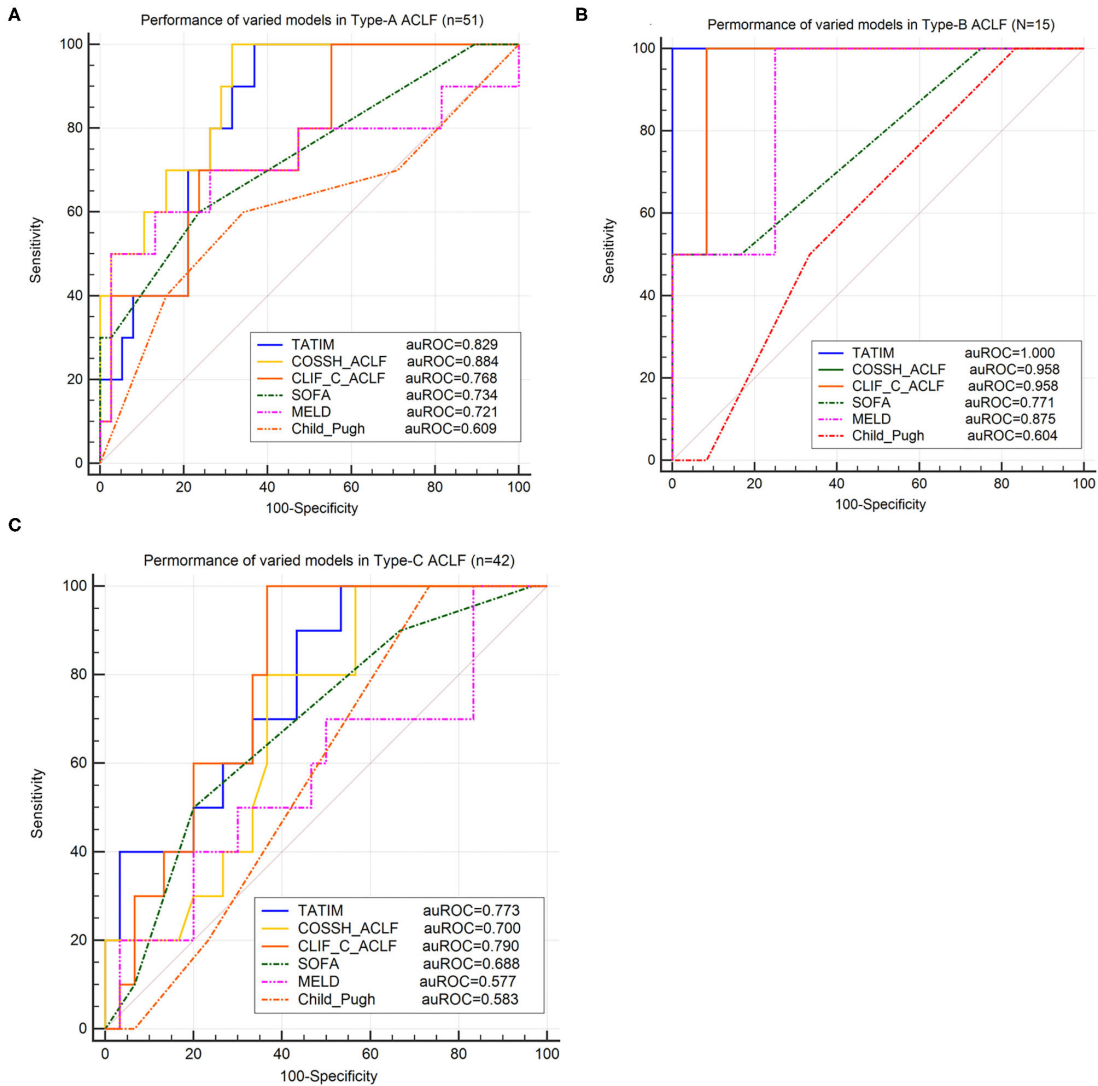
Validation of TATIM model in ACLF-subtypes. ROC analysis was conducted in three ACLF-subtypes (Total *n* = 108, Type-A *n* = 51, Type-B *n* = 15 and Type-C *n* = 42). **(A–C)** Compared the area under receiver operating characteristic cure (AUROC) of TATIM model and other prognostic scores for predicting 28day survival in ACLF-subtype A, B and C, respectively. (AUROC of type-A, type-B and type-C were 0.829, 1.000 and 0.773, respectively; all *P* < 0.001).

Regardless of the ACLF subtypes, TATIM score had high prediction accuracy for the 28-day mortality. The AUROCs of type-A (*n* = 51), type-B (*n* = 15) and type-C (*n* = 42) were 0.829 (*p* < 0.001), 1.000 (*p* < 0.001) and 0.773 (*p* < 0.001), respectively (Figures 6A–C). The sensitivity and specificity of Type-A were 1.00 and 0.641 at cut-off value −1.855, those of Type-B were 1.00 and 1.00 at cut-off value −0.131 and those of Type-C were 1.00 and 0.467 at cut-off value −2.092. Compared with CLIF-C ACLF score, TATIM score performed better in predicting the 28-day outcomes for subtype-A ACLF (AUROC = 0.829 vs. 0.768) and subtype-B ACLF (AUROC = 1.000 vs. 0.958). Compared with COSSH ACLF score, TATIM score performed better in predicting the 28-day outcomes for subtype-B ACLF (AUROC = 1.000 vs. 0.958) and subtype-C ACLF (AUROC = 0.773 vs. 0.700).

## Discussions

Early warning of ACLF is necessary and urgent. In clinical practice, not only the short-term mortality of ACLF patients, but also the disease progression of non-ACLF patients are supposed to be emphasized. For the patients who are diagnosed with ACLF at admission, mortality is deserved to be focused on, which guide medical staff to take aggressive and timely measures to save patients' lives. And for the baseline non-ACLF patients whose liver function have already been impaired, we need a timely judgment whether it will get worse or even develop into ACLF. This study explored in both of the two aspects and revealed the predictive ability of testosterone and estradiol respectively. Testosterone was a novel predictor of short-term mortality in HBV-ACLF irrespective of the ACLF subtypes, while estradiol did better in predicting the progression to HBV-ACLF.

A qualified prognostic model is supposed to meet three criteria. Firstly, it has a high accuracy, by which the short-term outcome can be predicted with higher sensitivity and specificity. TATIM model developed in this study has a good performance and its AUROC in predicting 28d mortality is higher than that of Child-Pugh, MELD, iMELD and SOFA and is comparable or even higher than that of CLIF-C ACLF and COSSH ACLF scores. Secondly, it is simple and practical. It is best to maximize the prediction with the least number of parameters and operations. TATIM model is simple, consisting of age, gender, TBil, INR and testosterone. This is in sharp contrast with Child-Pugh, SOFA, CLIF-SOFA, CLIF-C ACLF, COSSH-ACLF and others, which is involved in complex multi-system scoring. Thirdly, it has a wide suitability. Child-Pugh, MELD, iMELD, SOFA, CLIF-SOFA and CLIF-C ACLF were developed in the western cohort where FLD and ALD were dominated. TATIM model was designed in HBV-ACLF cohort and had good predicting performance in ACLF subtype A, B and C. The TATIM model fulfilled all the three requirements and had a promising prospect.

As for simple prognostic models, HINT model proposed in 2018 and P5 model proposed in 2020 are novel and efficient in predicting the short-term mortality of HBV-ACLF. HINT introduces thyroid-stimulating hormone (TSH) into the prognostic model for the first time. However, the reduction of TSH is a universal manifestation in patients with severe diseases (22) and the evidence of TSH's specific effect on ACLF is rare. Relevant studies suggested that the reduced thyrotropin -releasing hormone (TRH) may be a compensator response to the inflammatory cytokine induced by oxidative stress (26, 27), which is crucial in the pathogenesis of HBV-ACLF. The plasminogen mentioned in P5 score is another parameter involved in coagulation systems (25), which overlaps with INR to some extent. Likewise, it remains unclear how plasminogen interact with ACLF. The only evidence shows that plasminogen activation by the plasmin-α2-antiplasmin system plays a potential role in hepatocyte regeneration after damage (28) and decreased plasminogen levels would hamper liver's self-repairing capability (29). In spite of their high prediction accuracy in statistics, the mechanism underlying TSH and plasminogen remains further investigation.

As typical androgen and estrogen, testosterone and estradiol involving in this study have drawn extensive attention. Numerous studies show that gender disparity exist in many liver diseases such as NAFLD, alcoholic liver disease (ALD), CHB, autoimmune hepatitis (AIH) and HCC. In 1985, Nagasue observed serum levels of testosterone and estrogens in cirrhotic men with or without HCC and found the estrogen-testosterone ratio was significantly higher in HCC group (6), which raised the research trend of estrogen-carcinogenesis mechanism in liver. Grossman's research firstly showed the low testosterone levels were associated with poor prognosis in chronic liver diseases in 2012 (10) and 2016 (9) and Klair revealed that estrogen deficiency could increase fibrosis risk among postmenopausal women with NAFLD in 2016 (30). As for the underlying mechanism, Lee et al. and Kim et al. clarified that estrogen and estrogen-related receptor γ(ERRγ) affected the progression of ALD respectively via hepatic IκB (31) and CB1R-CYP2E1-mediated oxidative stress (15). Li et al. proved that Foxa1/a2 played essential roles in sexual dimorphism of HCC in 2012 (32) and Zhang et al. suggested that overexpression of nuclear androgen receptor driven by PI3K-AKT- mTOR pathway was associated with progression and prognosis of HCC in 2018 (33). For HBV-related HCC, additional evidence was found by Chiu et al. in 2007 and Yu et al. in 2014. Chiu et al. identified two conserved androgen response elements (ARE) with the enhancer I of HBV and suggested an AR-mediated stimulation of HBV transcription (13). Yu et al. found that cell cycle-related kinase (CCRK) mediated the HBx-AR signaling in viral-host oncogenic circuitry which induced hepatocellular proliferation and transformation (34). However, evidence of sex hormone's effects on HBV-related ACLF is rare and so is the research about mechanism.

It is the first time that the level of serum testosterone and estradiol were compared in patients with varied ACLF background, disease severity and cirrhosis conditions. Patients with severe clinical conditions tendered to have lower testosterone and higher estradiol levels irrespective of the history of liver cirrhosis or decompensation. Given the current understanding that sexual hormones are closely related with pro- and anti-inflammatory pathophysiological processes, further studies would spring up in the future.

This study had several limitations. Firstly, it lacks some longitudinal observations of sex hormone levels to evaluate the prognostic ability adequately. Secondly, multi-centered data are needed to make the new model more convincing. The specificity of TATIM model is 0.601, which is low and a verification data should be stronger and robust for the model. Thirdly, whether sex hormones can be prognostic predictor of ACLF caused by other etiologies still needs further investigations.

In conclusion, this study firstly highlighted the predictive ability of androgen and estrogen in the progression and prognosis of HBV-related ACLF. Moreover we developed a novel and simple prognostic model-TATIM, which had a high accuracy irrespective of the HBV-ACLF subtypes. We hope that the new findings will facilitate the clinical management of HBV-ACLF and enlighten researchers to explore the underlying mechanism.

## Data Availability Statement

The raw data supporting the conclusions of this article will be made available by the authors, without undue reservation.

## Ethics Statement

The studies involving human participants were reviewed and approved by Shanghai Jiaotong University School of Medicine, Renji Hospital Ethics Committee. The patients/participants provided their written informed consent to participate in this study.

## Author Contributions

GD and SS: study design. SS and BX: patient enrollment. SS, XX, WT, YZ, YD, and YG: data entry. SS and ZT: laboratory examination of samples. SS: statistical analysis and interpretation of data. SS and GD: drafting of the manuscript. GD: critical revision of the manuscript and study supervision. All authors contributed to the article and approved the submitted version.

## Funding

This study was supported in part by the National Natural Science Foundation of China (81930061, 81900579), the Chinese State Key Project Specialized for Infectious Diseases (2018ZX10723203), the National Science and Technology Major Project (2018ZX10732202), and the Chongqing Natural Science Foundation (CSTC2019jcyj-zdxmX0004).

## Conflict of Interest

The authors declare that the research was conducted in the absence of any commercial or financial relationships that could be construed as a potential conflict of interest.

## Publisher's Note

All claims expressed in this article are solely those of the authors and do not necessarily represent those of their affiliated organizations, or those of the publisher, the editors and the reviewers. Any product that may be evaluated in this article, or claim that may be made by its manufacturer, is not guaranteed or endorsed by the publisher.

## Abbreviations

HBV: hepatitis B virus
ACLF: acute-on-chronic liver failure
CHB: chronic hepatitis B
AUROC: area under receiver operating characteristic curve
MELD: Model for End-Stage Liver Disease
iMELD: the integrated MELD
SOFA: Sequential Organ Failure Assessment
CLIF-C: Chronic Liver Failure Consortium
EASL: European Association for the Study of the Liver
CANONIC: Consortium Acute-On-Chronic Liver Failure in Cirrhosis
COSSH: Chinese Group on the Study of Severe Hepatitis B
NAFLD: non-alcoholic fatty liver disease
HCC: hepatocellular carcinoma
OPLS-DA: Orthogonal Partial Least Squares Discrimination Analysis
CLD: chronic liver disease
UGIB: upper gastrointestinal bleeding
HE: hepatic encephalopathy
HRS: hepatorenal syndrome
ALT: alanine aminotransferase
AST: aspartate aminotransferase
TBil: total bilirubin
PT: prothrombin activity
CRF: case report form
CT: computerized tomography
HBeAg: hepatitis B e antigen
E2: estradiol
TT: testosterone
INR: international normal ratio
VIF: variance inflation factor
TSH: thyroid-stimulating hormone
TRH: thyrotropin-releasing hormone
ALD: alcoholic liver disease
AIH: autoimmune hepatitis.
