# Supplementary material for: Testosterone and Estradiol as Novel Prognostic Indicators for HBV-Related Acute-on-Chronic Liver Failure
Source: Front Med (Lausanne). 2021 Sep 8;8:729030. doi: 10.3389/fmed.2021.729030 (PMC8455926; doi:10.3389/fmed.2021.729030)
Supplement: Supplementary file 1 [file Table_1.DOCX]

**SUPPLEMENTARY TABLE 1**

**Characteristics of non-ACLF patients’ progression during hospitalization (within 28days)**

| **Variables** | **Total(n=192)** | **28day**  **new-ACLF (n=20)** | **28day**  **non-ACLF (n=172)** | **p value** |
| --- | --- | --- | --- | --- |
|  |  |  |  |  |
| **Clinical characteristics** | |  |  |  |
| Age (year) | 44.40±11.99 | 43.2±13.36 | 44.53±11.85 | 0.639 |
| Male, n (%) | 161(83.9) | 19(95.0) | 142(82.6) | 0.152 |
| Average Hospital Stay, (days) | 19.46±11.77 | 29.75±16.64 | 18.27±10.49 | <0.001 |
| Bacteria infection, n (%) | 37(19.3) | 6(30.0) | 31(18.0) | 0.199 |
| HBV reactivation, n (%) | 50(26.0) | 6(30.0) | 44(25.6) | 0.670 |
| Alcohol intaking, n (%) | 15(7.8) | 0(0) | 15(8.7) | 0.374 |
| Heptoxic drug, n (%) | 9(4.7) | 1(5.0) | 8(4.7) | 1.000 |
| Fatigue, n (%) | 6(3.1) | 0(0) | 6(3.5) | 1.000 |
| UGIB, n (%) | 3(1.6) | 0(0) | 3(1.7) | 1.000 |
| Cirrhosis, n (%) | 96(50.0) | 8(40.0) | 88(51.2) | 0.345 |
| Ascite, n (%) | 65(33.9) | 3(15.0) | 62(36.0) | 0.060 |
| HE, n (%) | 0(0) | 0(0) | 0(0) |  |
| HBeAg +, n (%) | 104(54.2) | 11(57.9) | 93(58.5) | 0.960 |
| **Laboratory parameters** | |  |  |  |
| WBC (10^9/L) | 5.08±2.82 | 5.19±2.34 | 5.07±2.87 | 0.857 |
| Neutrophil (10^9/L) | 3.54±3.65 | 3.64±1.69 | 3.53±3.91 | 0.898 |
| Leukomonocyte (10^9/L) | 0.38±0.46 | 0.45±0.29 | 0.37±0.48 | 0.473 |
| TBil (mg/dL) | 6.72±6.57 | 8.94±6.44 | 6.46±6.55 | 0.111 |
| ALT (IU/L) | 518.02±694.90 | 1254.12±1234.0 | 432.42±547.2 | 0.008 |
| AST(IU/L) | 387.95±448.53 | 742.65±525.31 | 346.71±421.45 | 0.004 |
| AKP(IU/L) | 134.26±50.97 | 144.06±46.27 | 133.1951.47 | 0.392 |
| Alb (g/L) | 33.62±10.32 | 34.69±5.33 | 33.50±10.73 | 0.635 |
| INR | 1.24±0.23 | 1.46±0.27 | 1.22±0.22 | <0.001 |
| Sodium (mmol/L) | 137.67±10.08 | 137.97±3.79 | 137.64±1.59 | 0.890 |
| Kalium (mmol/L) | 4.72±9.81 | 3.98±0.48 | 4.80±10.38 | 0.823 |
| Creatine (mg/dL) | 0.77±0.34 | 0.73±0.15 | 0.78±.36 | 0.536 |
| Log HBV-DNA(IU/ml) | 4.26±2.75 | 5.29±2.84 | 4.14±2.72 | 0.083 |
| **Sexual hormones** | |  |  |  |
| E2 (pg/ml) | 73.15±73.60 | 107.37±92.48 | 69.21±70.39 | 0.032 |
| TT (ng/ml) | 4.32±3.58 | 4.23±3.25 | 4.33±3.63 | 0.905 |
| **Scoring systems** | |  |  |  |
| Child-Pugh | 7.93±1.69 | 8.65±1.09 | 7.85±1.73 | 0.007 |
| MELD | 10.27±5.45 | 14.11±3.75 | 9.80±5.45 | 0.001 |
| iMELD | 2.35±0.69 | 2.76±0.64 | 2.30±0.68 | 0.004 |
| SOFA | 3.58±1.63 | 5.00±1.03 | 3.41±1.61 | <0.001 |
| CLIF-C ACLF | 28.76±7.12 | 29.70±8.34 | 28.65±6.99 | 0.534 |
| COSSH ACLF | 4.55±0.52 | 4.75±0.53 | 4.53±0.52 | 0.082 |
| TATIM | -6.82±4.15 | -6.39±4.08 | -6.87±4.17 | 0.638 |

AKP: phosphatase alkaline; ALT: alanine aminotransferase; AST: aspartate aminotransferase; CLIF‐C ACLF: European Association for the Study of Chronic Liver Failure; COSSH ACLF：ACLF model of Chinese Group on the Study of Severe Hepatitis B;; E2:estradiol; HBeAg : hepatitis B e antigen; HBV: hepatitis B virus; HE: hepatic encephalopathy; INR: international normalised ratio; MELD : Model for End‐stage Liver Disease; SOFA : sequential organ failure assessment; TT: testosterone; UGIB : upper gastrointestinal haemorrhage ; WBC: white blood count.
